# A Noninvasive Approach to Evaluate Tumor Immune Microenvironment and Predict Outcomes in Hepatocellular Carcinoma

**DOI:** 10.1007/s43657-023-00136-8

**Published:** 2023-12-08

**Authors:** Jianmin Wu, Wanmin Liu, Xinyao Qiu, Jing Li, Kairong Song, Siyun Shen, Lei Huo, Lu Chen, Mingshuang Xu, Hongyang Wang, Ningyang Jia, Lei Chen

**Affiliations:** 1 Shanghai Key Laboratory of Metabolic Remodeling and Health, Institute of Metabolism and Integrative Biology, Fudan University, Shanghai, 200438 China; 2 The International Cooperation Laboratory on Signal Transduction, Eastern Hepatobiliary Surgery Hospital, Naval Medical University, Shanghai, 200438 China; 3 National Center for Liver Cancer, Shanghai, 201805 China; 4 Department of Radiology, Tongji Hospital, School of Medicine, Tongji University, Shanghai, 200333 China; 5 Department of Oncology, Shanghai Medical College, Fudan University, Shanghai, 200032 China; 6 Department of Radiology, Union Hospital, Tongji Medical College, Huazhong University of Science and Technology, Wuhan, 430022 China; 7 Department of Radiology, Third Affiliated Hospital of Naval Medical University, Shanghai, 200438 China

**Keywords:** Hepatocellular carcinoma, Tumor immune microenvironment, Radiomic, Prognosis, Immunotherapy response

## Abstract

It is widely recognized that tumor immune microenvironment (TIME) plays a crucial role in tumor progression, metastasis, and therapeutic response. Despite several noninvasive strategies have emerged for cancer diagnosis and prognosis, there are still lack of effective radiomic-based model to evaluate TIME status, let alone predict clinical outcome and immune checkpoint inhibitor (ICIs) response for hepatocellular carcinoma (HCC). In this study, we developed a radiomic model to evaluate TIME status within the tumor and predict prognosis and immunotherapy response. A total of 301 patients who underwent magnetic resonance imaging (MRI) examinations were enrolled in our study. The intra-tumoral expression of 17 immune-related molecules were evaluated using co-detection by indexing (CODEX) technology, and we construct Immunoscore (IS) with the least absolute shrinkage and selection operator (LASSO) algorithm and Cox regression method to evaluate TIME. Of 6115 features extracted from MRI, five core features were filtered out, and the Radiomic Immunoscore (RIS) showed high accuracy in predicting TIME status in testing cohort (area under the curve = 0.753). More importantly, RIS model showed the capability of predicting therapeutic response to anti-programmed cell death 1 (PD-1) immunotherapy in an independent cohort with advanced HCC patients (area under the curve = 0.731). In comparison with previously radiomic-based models, our integrated RIS model exhibits not only higher accuracy in predicting prognosis but also the potential guiding significance to HCC immunotherapy.

**Supplementary Information:**

The online version contains supplementary material available at 10.1007/s43657-023-00136-8.

## Introduction

A growing number of studies have shown that tumor immune microenvironment (TIME) is associated with prognosis, progression, metastasis, and therapeutic response (Abdul Sater et al. 2020; Binnewies et al. 2018; Fridman et al. 2017). Immune checkpoint inhibitors (ICIs) using antibodies against programmed cell death 1 (PD-1) or programmed death ligand 1 (PD-L1) had achieved great success in cancer treatment, meanwhile not all patients responded to the ICIs immunotherapy (Xu et al. 2022). Retrospective analyses of patient populations treated with ICIs have revealed that complicated heterogeneity of the intra-tumoral immune microenvironment contributes largely to distinct response and tumor progression after treatment (Binnewies et al. 2018; Fridman et al. 2017). Currently, the most commonly used method for assessing the immune status of tumor patients is the immune score, which was applied to predict clinical outcomes in patients with cancer (Galon et al. 2006; Mlecnik et al. 2011). But previous evaluations of immune score was mostly based on multiple sections of immunohistochemistry or only a single or a small number of immune cells, which cannot provide comprehensive and detailed information for TIME evaluation, and biopsies are needed to evaluate the immune score, which not only caused trauma to the patients but also had the potential to promote tumor metastasis. It would be valuable to develop a noninvasive method to evaluate TIME, and it is also critical to predict immunotherapy response through TIME value.

Medical imaging plays a vital role in the diagnosis of diseases since its discovery, especially in oncologic diagnosis and treatment guidance (Aerts et al. 2014). As technology has advanced, the image provided us with more information than before. The so-called radiomics was emerging during these years. Based on the image-based signature, radiomics can achieve precision diagnosis and give treatment guidance (Lambin et al. 2017). Recent studies have revealed that radiomic signature was able to estimate the abundance of cluster of differentiation 8 (CD8) cells inside tumor, discriminate inflamed tumors from immune-desert tumors and predict response to anti-PD-1 and anti-PD-L1 immunotherapy (Sun et al. 2018). Computed tomography (CT)-based Radiomic Score was related to the neutrophil-to-lymphocyte ratio (NLR) in the TIME, and the Radiomic Score was correlated with prognosis and immunotherapy response in advanced gastric cancer patients (Huang et al. 2022). According to previous studies, radiomic features were associated with macrovascular invasion (Xu et al. 2019), prognosis (Xu et al. 2019; Zhang et al. 2020), pathologic grade (Wu et al. 2019), and recurrence (Zhao et al. 2021; Zhou et al. 2017). Radiomic model was also used to predict the protein expression inside tumor (Tian et al. 2021; Wang et al. 2020; Yang et al. 2021). Other studies had shown that radiomic model was able to predict the treatment response of several cancers such as rectal cancer (Blazic et al. 2017; Liu et al. 2017; Nie et al. 2016), cervical cancer (Lucia et al. 2018), glioblastoma (Kickingereder et al. 2016), gastric cancer (Jiang et al. 2020), and hepatocellular carcinoma (HCC) (Yuan et al. 2020).

HCC is a highly malignant cancer and it becomes the fourth most common cause of cancer-related death in the world (Foerster et al. 2022; Yang et al. 2019a). The liver is an important and critical component in the defense against blood-borne infection, for its receiving both vein blood and arterial blood, and it is continuously exposed to blood-borne pathogens; thus, it has a plethora of innate and adaptive immune cells (Jenne and Kubes 2013). The balance of the microenvironment is critical and it plays an important role in HCC development (Makarova-Rusher et al. 2015). For example, the enrichment of CD8 + T lymphocytes in TIME is associated with a better prognosis (Flecken et al. 2014; Garnelo et al. 2017). At present, the major treatment options for very early stage and early stage HCC are surgical resection, ablation, and transplantation (Llovet et al. 2021). Over the past decades, immunotherapy has been widely used in the treatment of HCC. Immune checkpoint inhibitors (ICIs) like atezolizumab and bevacizumab have become the first-line therapeutic method for advanced HCC (Foerster et al. 2022). But not all patients benefit from ICIs. Nivolumab, an anti-PD-1 antibody, has achieved an overall response rate of 14% with a median response duration of 17 months (El-Khoueiry et al. 2017). To date, there are no robust biomarkers predicting response to ICIs in patients with HCC (Llovet et al. 2022). The clinical features, PD-L1 expression, gene-expression profiling, and gut microbial diversity were used to predict response to ICIs (El-Khoueiry et al. 2017; Haber et al. 2023; Hu et al. 2019; Sangro et al. 2020; Yu et al. 2021; Zheng et al. 2019). For clinical features and gut microbial, they are lack of accuracy, and for PD-L1 expression and gene-expression profiling, they are invasive methods and might cause metastasis. Therefore, a noninvasive and reliable method needs to be developed.

Here we developed a new method to evaluate TIME within tumor and predict outcomes in patients with HCC. We detected 17 immune-related protein markers expression inside tumor. TIME value was evaluated by the expression of immune-related markers. Afterward, we developed a radiomic model using a machine learning method to predict the TIME value and investigate its potential of predictive power for prognosis and anti-PD-1 immunotherapy response.

## Materials and Methods

### Patients and Specimens

**Fig. 1:**
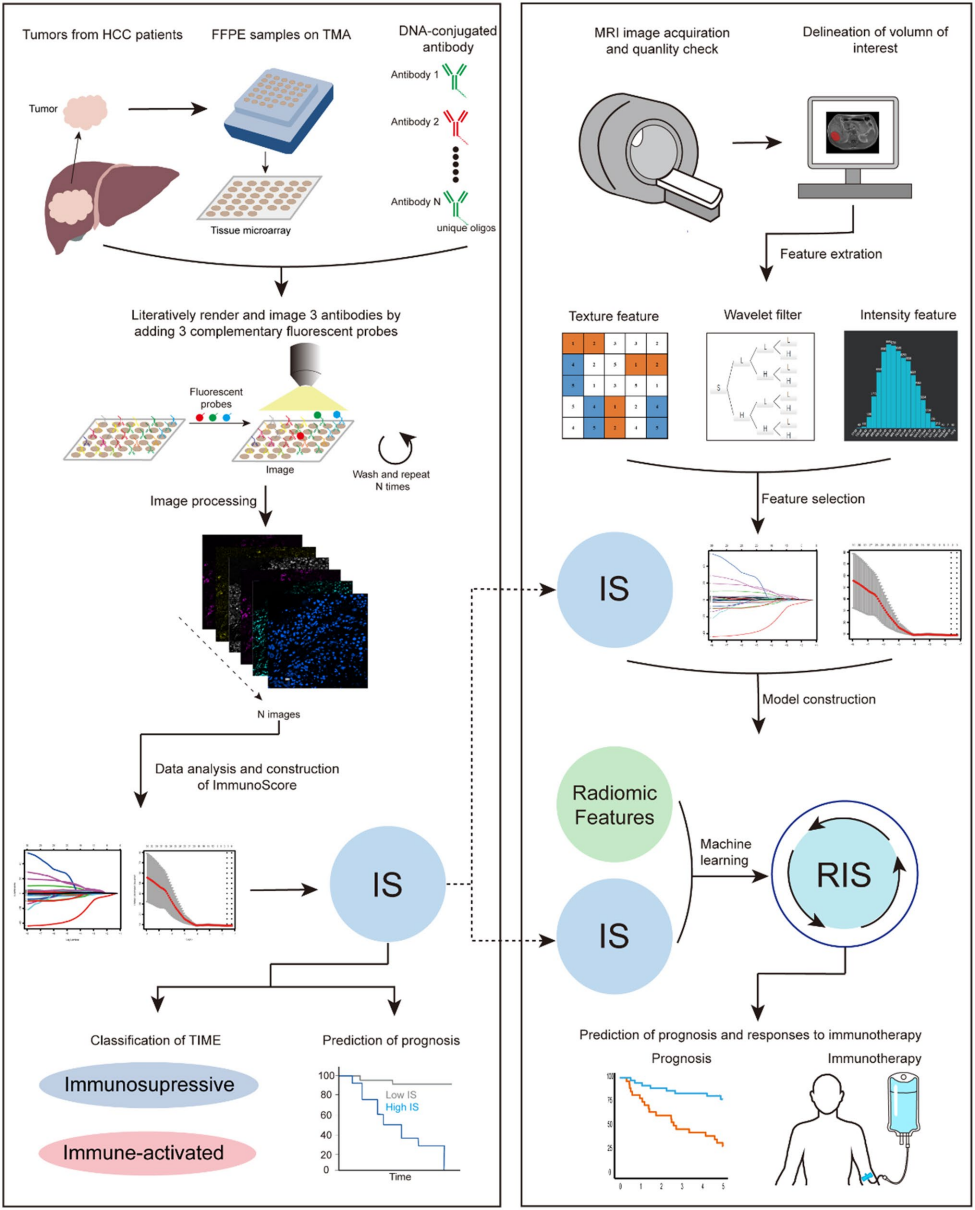
The establishment of radiomic immunoscore (RIS). Tissue microarray was incubated with 17 immune-related markers, and CODEX image was generated through image processing. Immunoscore (IS) was constructed based on the expression of immune-related markers. A predictive model for IS, referred as RIS was developed. The RIS was found to be associated with prognosis and could provide guidance for immunotherapy

The overall study design is shown in Fig. 1 and Fig. S1. The study was approved by Ethical Committee of Eastern Hepatobiliary Surgery Hospital (EHBH) (EHBHKY2018-1–001) and Union Hospital Tongji Medical College in Huazhong University of Science and Technology (2020–0151-01). A total of 487 patients were recruited in our study. A written informed consent was provided by every participant. After filtering, a total of 301 HCC patients in six independent cohorts were enrolled in this study (Table 1), including 258 male and 43 female cases. EHBH cohort 1 included 65 patients, EHBH cohort 2 included 104 patients, EHBH cohort 3 included 27 patients, and EHBH cohort 4 included 45 patients, The Cancer Genome Atlas (TCGA) cohort included 27 patients, immunotherapy cohort included 35 patients. Patients in EHBH cohort 1 and EHBH cohort 3 were available with co-detection by indexing (CODEX) data and magnetic resonance imaging (MRI) images, and patients in EHBH cohort 3, EHBH cohort 4, TCGA cohort, and immunotherapy cohort were available with MRI images. Patients in immunotherapy cohort were treated with anti-PD-1 antibody immunotherapy. Detailed clinicopathological characteristics are shown in Table 1 and Table S1.

**Table 1:** Clinical characteristics of patients in all cohorts

Variables	EHBH cohort 1 (*n* = 65)	EHBH cohort 2 (*n* = 104)	EHBH cohort 3 (*n* = 27)	EHBH cohort 4 (*n* = 45)	TCGA cohort (*n* = 25)	Immunotherapy cohort (*n* = 35)
Gender						
Female	10	12	1	5	12	2
Male	55	92	26	40	13	33
Age						
> 60	26	32	2	27	12	6
≤ 60	39	72	25	18	13	29
Stage						
I	48	93	17	36	6	1
II	2	0	4	5	8	1
III	5	7	0	3	8	15
IV	10	4	6	1	1	17
Unknown	0	0	0	0	2	1
Metastasis						
M0	55	100	21	44	20	16
M1	10	4	6	1	1	17
Mx	0	0	0	0	4	2

The inclusion criteria of HCC patients were as follows: (1) qualified MRI images and CODEX images; (2) MRI images were collected before treatment; for patients treated with anti-PD-1 immunotherapy, MRI images were collected before and after treatment; (3) complete follow-up records. Finally, a total of 301 HCC patients were enrolled in our study (Table 1; Fig. S2). Among them, 241 patients were treated with surgical resection at Eastern Hepatobiliary Surgery Hospital (Shanghai, China) from January 2010 to May 2016. Among them, 92 patients had both CODEX images and MRI images. In TCGA cohort, all of the patients received pharmaceutical therapy and radiation therapy. In immunotherapy cohort, all of the patients received PD-1 blockade. The PD-1 blockade used in the study included pembrolizumab (200 mg IV every 21 days), camrelizumab (200 mg IV every 21 days), tislelizumab (200 mg IV every 21 days), and sintilimab (200 mg IV every 21 days).

We used computer-generated random numbers to assign 65 patients (EHBH cohort 1) to the training cohort and 27 patients (EHBH cohort 3) to the testing cohort. One hundred and four patients (EHBH cohort 2) are available with CODEX images, and 45 patients (EHBH cohort 4) are available with MRI images. Twenty-five patients from 2003 to 2013 (TCGA cohort) were collected from the databases of TCGA (http://tcgaportal.org/) and The Cancer Imaging Archive (TCIA, https://www.cancerimagingarchive.net/), MRI images were available. Thirty-five patients (immunotherapy cohort) treated with anti-PD-1 immunotherapy from September 2019 to May 2022 were collected from Union Hospital Tongji Medical College Huazhong University of Science and Technology, MRI images were available.

### Construction of Immunoscore (IS)

We constructed IS based on CODEX images of tumor area from each patient. The CODEX work flow is shown in Fig. 1. CODEX was a commercialized and accessible multiplexed tissue imaging platform (Akoya Biosciences, Menlo Park, California, USA) which was developed by Garry P. Nolan and his colleagues (Schurch et al. 2020). The CODEX technology uses oligonucleotide-conjugated antibodies and sequential fluorescent reporters, which can detect up to 60 protein markers simultaneously in a single tissue (Phillips et al. 2021). Formalin-fixed, paraffin embedded (FFPE) tissue and tissue microarrays (TMAs) were used in our study. Tissue samples were obtained from HCC patients treated at Eastern Hepatobiliary Surgery Hospital. Written informed consent was obtained from all patients.

We selected 17 immune-related biomarkers for CODEX staining: Foxp3, PD-L1, PD-1, CD163, CD45, CD45RO, CD107A, CD21, CD68, CD8, CD3, CD4, CD11C, human leukocyte antigen DR (HLA-DR), CD44, CD20, and CD31. Among these biomarkers, some are biomarkers for cells, for example: Foxp3 (regulatory T cells), CD8 (cytotoxic T cells), CD20 (B cells); some biomarkers are expressed in a variety of cells: PD-L1, PD-1, HLA-DR, CD44, CD31. PD-L1 and PD-1 were regarded as immune check points (Chiu et al. 2020; Tichet et al. 2023); HLA-DR is an isotype of human leukocyte antigen; CD44 is a tumor biomarker and the expression of CD44 is related with tumor initiation and progression (Xu et al. 2020). CD31 is mainly expressed on the junctions of confluent endothelial monolayers (Paddock et al. 2016). The antibodies panel is shown in Table S2. CODEX processor software (Akoya Biosciences, version 1.7) was used to process the CODEX images. CODEX Multiplex Analysis Viewer (Akoya Biosciences, version 1.5.0.8) was used to analyze the protein expression inside each tumor. All of the markers were normalized by the CODEX Multiplex Analysis Viewer.

Among the seventeen immune-related molecules, we used least absolute shrinkage and selection operator (LASSO) Cox regression model with tenfold cross-validation to select the most useful prognostic features. The 'glmnet' package was used to perform the LASSO Cox regression model analysis. Complete details are provided in Supplementary Materials.

### Radiomics Workflow

The radiomics workflow is shown in Fig. 1. All tumors were manually delineated by reader 1 (KS, with a 6-year work experience in liver imaging), reader 2 (WL, with a 5-year work experience), and reader 3 (JL, with an 8-year work experience) on the T1-weighted images, T2-weighted images, diffusion-weighted images with b values of 600 s/mm^2^, enhanced arterial phase, portal venous phase and delayed phase using ITK-SNAP software (Version 3.6). Reader 4 (NJ, with 20 years of experience in liver imaging) independently performed the segmentation to evaluate test–retest and inter-reader reproducibility, respectively. The reproducibility was subject to the intraclass correlation coefficient.

Feature extraction and image preprocessing were performed with the 3D Slicer software (version 4.9.0). Images were resampled to a voxel size of 1 × 1 × 1 mm to standardize the voxel spacing; voxel intensity values were discretized using a fixed bin width of 25 HU to reduce image noise and normalize intensities, allowing for a constant intensity resolution across all tumor images. We extracted 1223 radiomic features from each three-dimensional segmentation, giving a total of 6115 features for every lesion.

### Construction of Radiomic Immunoscore (RIS)

Among 6115 features, the coefficient of variation (CV) of each feature was evaluated, and then Mann–Whitney test, LASSO method with tenfold cross-validation was used to select the predictive radiomic features from EHBH cohort 1 (Fig. S5a, b). Finally, five predictive radiomic features were filtered out (Table S3). The RIS was built using ridge regression model. The optimal cutoff value for RIS was determined by Youden’s index in the training cohort.

### Clinical Data

Clinical and laboratory data were collected from electronic patient records, including age, gender, tumor node metastasis (TNM) stage, and metastasis. Tumor staging stratification was performed on the basis of the American Joint Committee on Cancer TNM Staging Manual, 8th edition (Chun et al. 2018). Outcome data included overall survival and clinical benefit. Overall survival was defined as the time to death from any cause within five years after treatment. Clinical benefit was defined as stable disease (SD) after five months of treatment or partial response (PR) or complete response (CR) within the first five months of treatment according to RECIST 1.1 (Eisenhauer et al. 2009).

### Statistical Analysis

We compared two groups by performing Student's *t* test for continuous variables. Survival curves were generated according to the Kaplan–Meier method and compared using the log-rank test. Univariate and multivariate analyses were performed using the Cox proportional hazards model. *p *value < 0.05 was considered as significant in two-tailed and one-tailed analyses. The area under the curve (AUC) and 95% CI (DeLong method) were determined from the receiver operating characteristic curve. The logistic and Cox regression coefficients were generated to generate nomograms. Calibration plots were generated to explore the performance characteristic of the nomograms. Nomograms and calibration curves were generated using ‘rms’ package with R software (version 3.6.3). Statistical analyses were done with R software (version 3.6.3).

## Results

### Evaluation of TIME Value

**Fig. 2:**
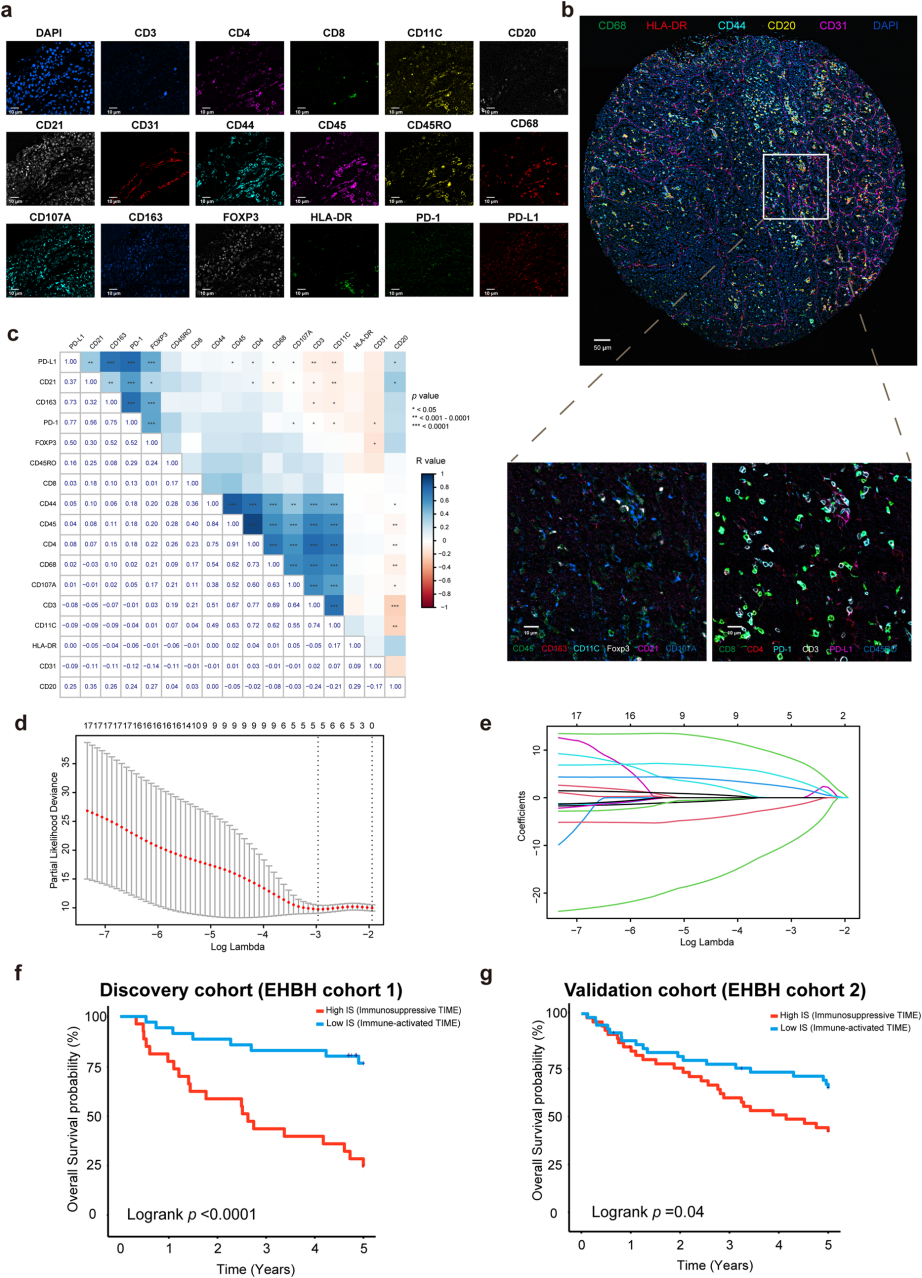
Construction and prognosis value of IS. **a** Images of a single tissue region color for each antibody. Scale bar, 10 um. **b** Spatial interaction of each immune-related markers. **c** Correlation between 17 immune-related markers. **d** Tuning parameter (λ) selection in the LASSO model used via tenfold cross-validation. **e** LASSO coefficient profiles of the 17 markers. **f** Kaplan–Meier analyses of OS according to IS signature in discovery cohort (EHBH cohort 1). **g** Validation cohort (EHBH cohort 2)

We detected 17 immune-related markers inside tumor using CODEX technology (Fig. 2a). The relative spatial interaction of each protein marker is shown in Fig. 2b. Among all patients with CODEX data, a positive correlation between the expression of CD45 and CD44, CD4 and CD45 inside tumor was observed (*R* > 0.9, *p* < 0.0001) (Fig. 2c). Using EHBH cohort 1 as discovery cohort, we first used LASSO Cox regression model with tenfold cross-validation to build a prognostic classifier, which includes five immune-related markers (CD68, HLA-DR, CD44, CD20, CD31) out of 17 immune-related markers (Fig. 2d, e). Then we developed IS using these five immune-related markers to evaluate TIME. Patients were classified as high IS and low IS according to the optimum cutoff 0.672 determined by 'survminer' package in EHBH cohort 1. We found that the expression of PD-1 was significantly higher in the high IS group than that in the low IS group (*p* value < 0.05) (Fig. S3). Accordingly, we defined the high IS group as immunosuppressive TIME, and the low IS group as immune-activated TIME. We also found in immunosuppressive TIME, the expression of CD20, CD31, CD44, and CD163 was relatively higher than in immune-activated TIME (*p* value < 0.05) (Fig. S3).

We compared the overall survival in high IS group and low IS group. The 5-year overall survival (OS) in the high IS group was 28.6%, while in low IS group was 78.4% in EHBH cohort 1 (Fig. 2f). We then checked the IS performance with an independent validation cohort (EHBH cohort 2). The 5-year OS in high IS group was 44.0% while in low IS group was 63.0% in EHBH cohort 2 (Fig. 2g). Multivariate Cox regression analysis adjusting for clinicopathological variables further confirmed the IS as an independent prognostic factor for OS prediction (Tables S4, S6–S8). IS was also associated with TNM stage of HCC patients, IS of patients in stage III and stage IV was significantly higher than that of the patients in stage I and stage II (*p* value < 0.05) (Fig. S4a). In addition, the value of IS was significantly higher in patients with metastasis than that in non-metastasis patients (*p* value < 0.05) (Fig. S4b), which indicated that the state of TIME changes as metastasis occurs.

### Development and Validation of RIS

**Fig. 3:**
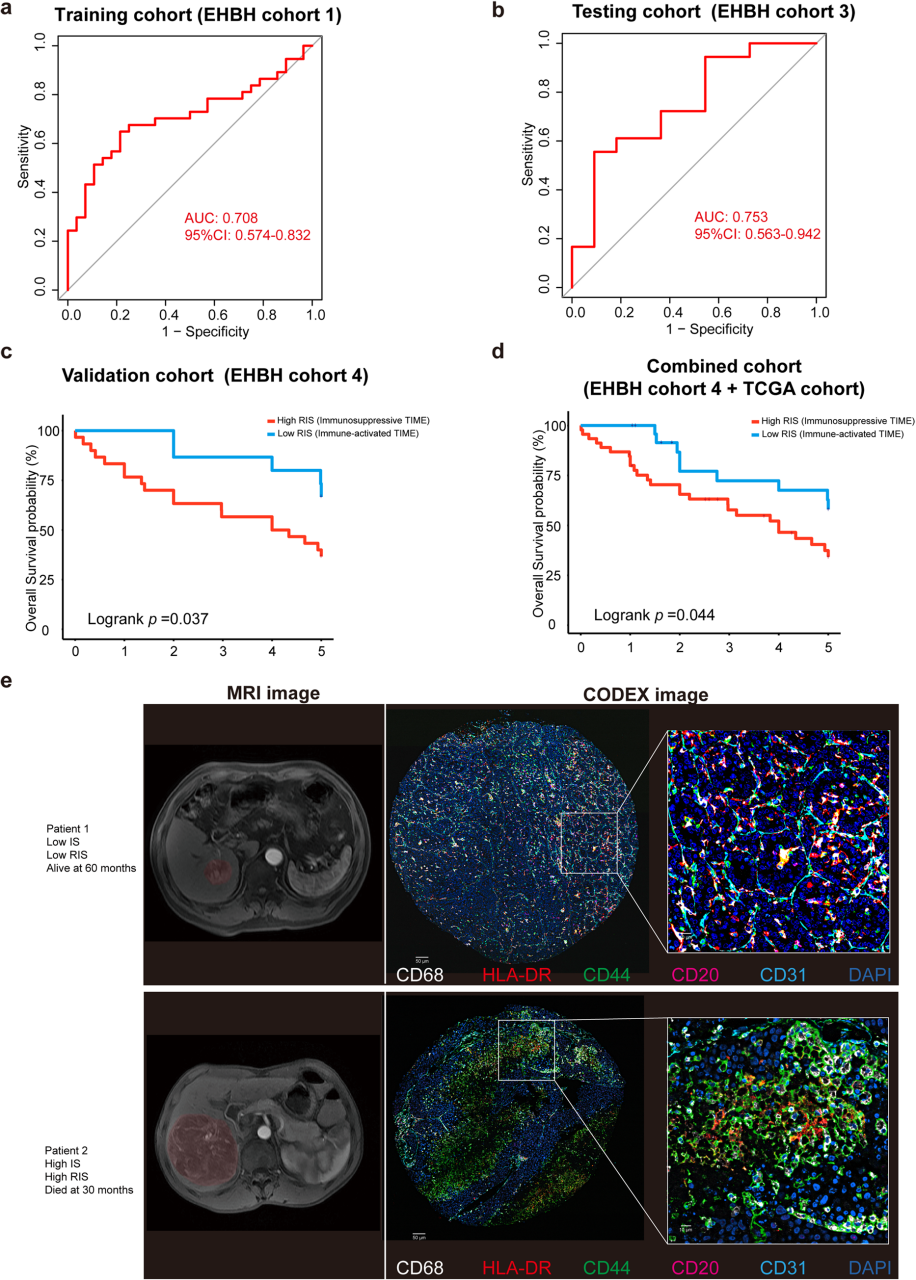
Performance of RIS in predicting TIME and prognosis in HCC patients. **a** Receiver operating characteristic curves of RIS in training cohort (EHBH cohort 1). **b** Testing cohort (EHBH cohort 3). **c** Kaplan–Meier analyses of overall survival of RIS in EHBH cohort 4. **d** Combined cohort. **e** Representative patients with immune-activated TIME and immunosuppressive TIME, along with their MRI images and CODEX images

Based on the extracted features of MRI images from HCC patients, we developed a radiomic model to predict IS. Among 6115 radiomic features, we finally filtered out five predictive radiomic features by the LASSO method and tenfold cross-validation (Fig. S5a, b). The five predictive radiomic features: Maximal correlation coefficient (MCC) of glcm-T1WI (LHH filtered), Contrast of glcm-T1WI (HLH filtered), GrayLevelVariance of glrlm-T1WI (HHL filtered), RunVariance of glrlm-Delayed phase (LHH filtered), and RunVariance of glrlm-Delayed phase (HLH filtered). Based on these predictive features, a total of six predictive models were tested, including the logistic regression model, support vector machines (SVM), ridge regression model, random forest, extreme gradient boosting (XGBoost), and linear regression model in the testing cohort, AUC was used to evaluate the performance of the model. Finally, we chose ridge regression model for its best performance in the testing cohort (Fig. S5c). The optimum cutoff of RIS was 0.426, determined using Youden's index in the training cohort. Accordingly, patients were classified into high RIS group (RIS ≥ 0.426) and low RIS group (RIS < 0.426). The ability of RIS to classify immunosuppressive TIME versus immune-activated TIME was shown to have an AUC of 0.708 [95% confidence interval (CI) 0.574–0.832] in the training cohort (EHBH cohort 1) (Fig. 3a), meanwhile it was 0.753 (95% CI 0.563–0.942) in the testing cohort (EHBH cohort 3) (Fig. 3b).

### Prognostic Value of RIS

**Fig. 4:**
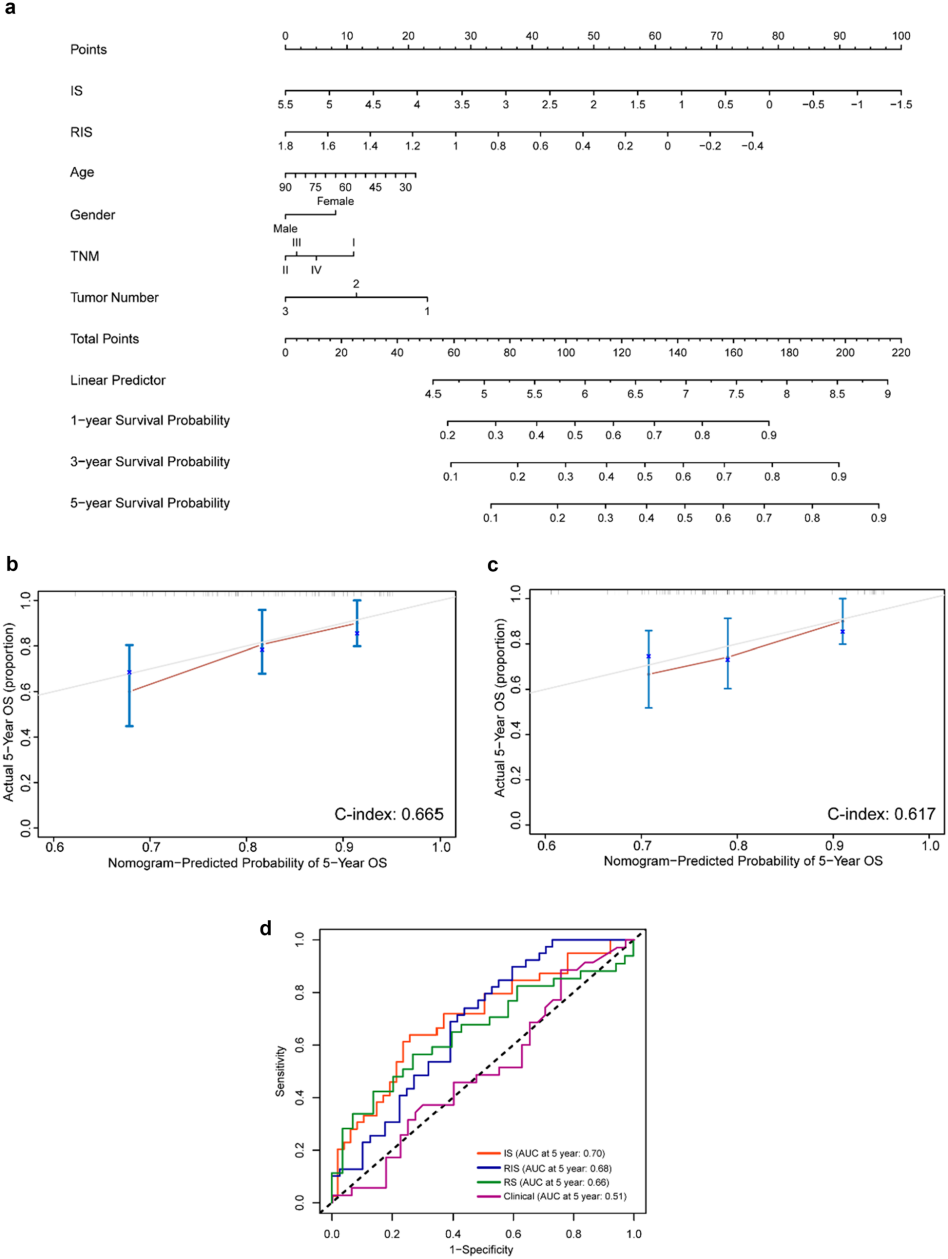
Nomogram based on RIS and clinicopathological factors and their calibration curve. **a** Nomogram predicting survival of HCC patients was established based on RIS and clinicopathological factors. **b** Calibration curves based on RIS for 5-year OS. **c** Calibration curves for 5-year OS based on RS and clinicopathological factors. **d** Time-dependent ROC for IS, RIS, clinical model, and radiomic model (RS)

We further assessed the prognostic value of RIS in an independent validation cohort (EHBH cohort 4). For the low RIS group, the overall 5-year survival was 64.3% whereas it was 38.7% for the high RIS group (Fig. 3c). In combined cohort (EHBH cohort 4 plus TCGA cohort), we observed the overall survival rate was 62.5% in low RIS group and 43.5% in high RIS group (Fig. 3d). Representative patients with immune-activated TIME and immunosuppressive TIME with their MRI images and CODEX images are shown in Fig. 3e. By performing multivariate Cox regression analysis adjusting for clinicopathological variables, the RIS was found as an independent prognostic factor for predicting OS (Tables S5–S10). An integrated model combing radiomics, clinical, and pathologic features consistently improved prognostic accuracy. A nomogram predicting the survival of HCC patients was established based on RIS, IS, and other clinicopathological factors (Fig. 4a). In addition, 500-sample bootstrapped calibration plot revealed the good predictive accuracy of the nomogram for the prediction of a 5-year survival rate (Fig. 4b). C-index was used to evaluate the predictive accuracy of the RIS-based nomograms, which was 0.646.

We also built a radiomic model based on the clinical data and radiomic features of the patients, called Radiomic Score (RS). LASSO-logistic regression model was used to filter out radiomic features. Then an integrated model was built based on RS, clinical, and pathologic features, a nomogram predicting the survival of HCC patients was established based on RS and clinicopathological factors (Fig. S6). 500-sample bootstrapped calibration curve was also plotted and C-index was 0.617 (Fig. 4c). We also compared the predictive effects of IS, RIS, clinical model, and radiomic model (RS) (Fig. 4d), and the construction of clinical model and radiomic model is shown in Supplementary Material.

### Predictive Value of RIS for Anti-PD-1 Immunotherapy Response

**Fig. 5:**
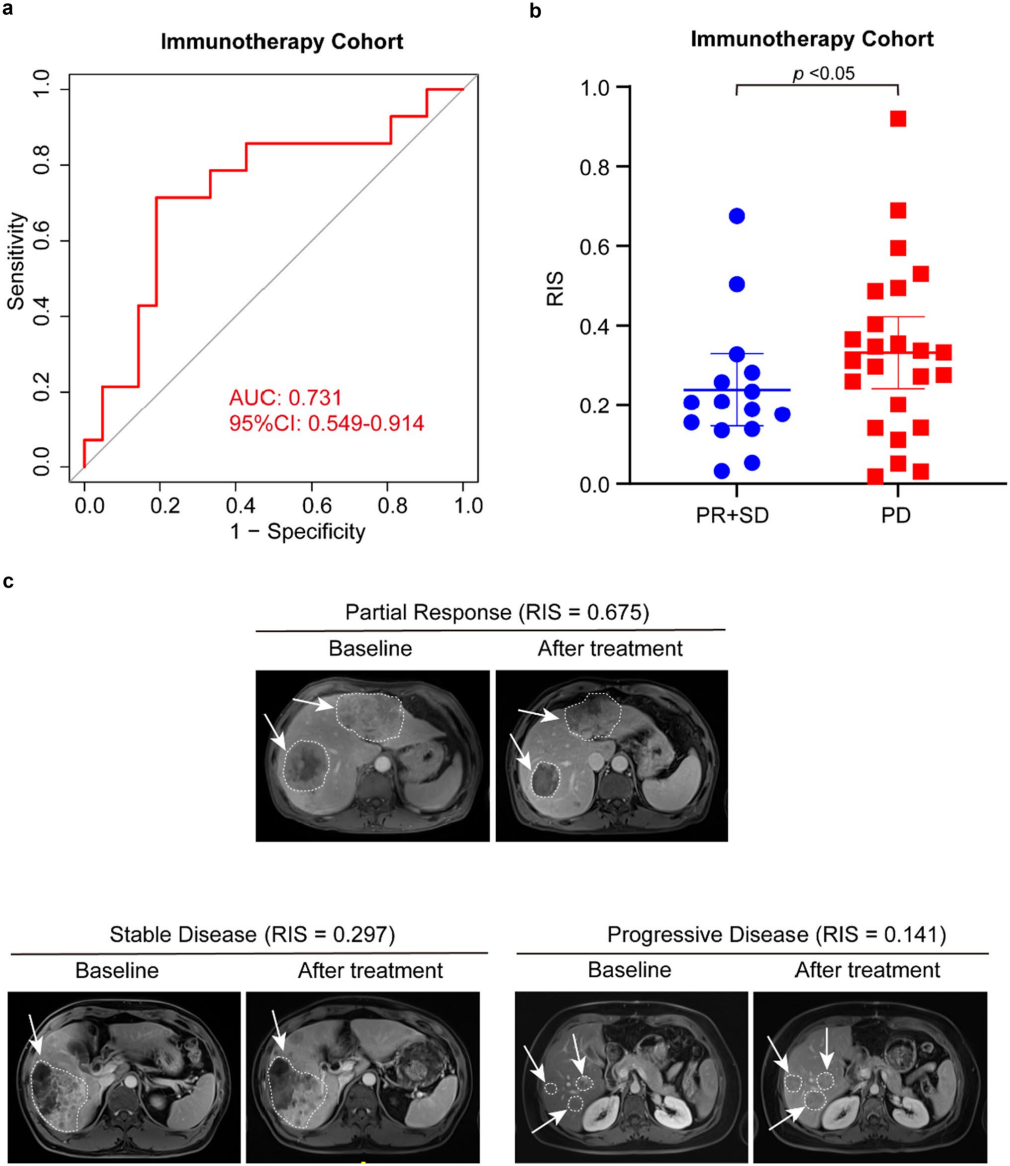
The association between RIS and responses to anti-PD-1 immunotherapy. **a** ROC curves of RIS for predicting response to anti-PD-1 immunotherapy. **b** RIS distribution in groups of clinical benefit patients and disease progressed patients. **c** RIS and MRI images of patients with different responses to anti-PD-1 immunotherapy

To further assess the potential predictive value of RIS, we evaluated the association between RIS and the response to anti-PD-1 immunotherapy response for HCC patients. Thirty-five patients who were treated with anti-PD-1 antibody were enrolled in our study. After treatment, 12 patients were defined as partial response (PR), nine patients were defined as stable disease (SD), and 14 patients were defined as progressive disease (PD) (Fig. 5c). Then we analyzed the association between RIS and responses to anti-PD-1 blockade. In terms of classification performance, the AUC was 0.731 (95%CI 0.549–0.914) (Fig. 5a). In addition, patients in low RIS group benefit more from anti-PD-1 immunotherapy than high RIS group (Fig. 5b), implying that RIS model might be a practical strategy to predict immunotherapeutic response in advanced HCC patients. Since IS is associated with TNM stage, we also explore the relation between TNM stages and responses to immunotherapy (Fig. S7). We found that there was no strong relation between TNM stage and response to immunotherapy in immunotherapy cohort.

## Discussion

TIME plays an important role in determining the prognosis and therapeutic effect in different types of cancers, including gastric cancer (Jiang et al. 2020), ovarian cancer (Zhang et al. 2003), colorectal cancer (Galon et al. 2006), and HCC (Wei et al. 2022). In our study, we detected 17 immune-related markers inside the tumor of HCC patients, then we found CD44, CD20, CD68, HLA-DR, and CD31 as the prognosis-related markers using LASSO Cox regression model. According to previous studies, CD44 is upregulated in HCC patients and has shown to positively correlate with poor prognosis and reduced patient survival (Dhar et al. 2018; Endo and Terada 2000). CD20 expressed in B cells and CD20 + B cells are positively correlated with the oncological prognosis of cholangiocarcinoma (Liu et al. 2022). CD68 is the marker of macrophages and elevated levels of CD68 were significantly related to poor overall survival in liver cancer (Wei et al. 2019; Zhang et al. 2022). Tumor HLA-DR expression is linked to early intrahepatic recurrence of HCC and low level of HLA-DR is associated with advanced tumor stage (Matoba et al. 2005). CD31 is the marker of endothelial cells, and the expression of CD31 was upregulated in tumor tissues (Hectors et al. 2020; Zhu et al. 2021). In our study, we found the expression of CD20, CD31, CD44, and CD163 was relatively higher in immune-activated TIME, which means those markers might be the activators in TIME. Some studies report the association between those markers and TIME, for instance, CD20 was the marker of B cell, and B cell was associated with tertiary lymphoid structures (Cao et al. 2023). Tumor-infiltrating B cells and plasma cells can serve as predictors of response to immune checkpoint inhibitors (Laumont and Nelson 2023). Research found that CD31 was an independent prognostic factor for HCC patients (Liu et al. 2021). CD44 is a malignant biomarker and it was upregulated in HCC cell lines and also in tumors (Kim et al. 2017). CD163 was a marker of M2-macrophage and an elevated level of CD163 is associated with poor prognosis in patients with small cell lung cancer (Klein et al. 2023).

In our study, we developed IS to evaluate TIME. We classified TIME as immunosuppressive and immune activated according to the level of IS and PD-1 expression. We found the immunosuppressive TIME was significantly related to poor overall survival in HCC patients. Based on patients' MRI radiomic features, we developed a radiomic prediction model to predict TIME status. The RIS was validated to have a superior performance to predict the prognosis of HCC patients. Since TIME status is associated with the effect of ICIs immunotherapy (Fridman et al. 2017), RIS was also validated as a predictor of responsiveness in ICIs immunotherapy.

There was a lot of research focusing on radiomic-based predicting model to predict TIME (Jiang et al. 2020; Perrone et al. 2022; Sun et al. 2018; Zheng et al. 2022), microvascular invasion (Yang et al. 2019b), the grade of HCC (Wu et al. 2019), recurrence (Wen et al. 2021), and prognosis of HCC (Long et al. 2019). In one study, Immunoscore was developed based on 27 immune features (Jiang et al. 2018), and later predicted Immunoscore based on CT images (Jiang et al. 2020). In another study, a radiomic model was developed to predict intratumor CD8 cells and response to immunotherapy and overall survival (Sun et al. 2018). In our research, we first developed an integrated model to predict both TIME and clinical outcomes simultaneously based on radiomic features. As compared with the RS model developed with radiomic data, RIS model obtained better performance and further exhibited its potential application for predicting immunotherapeutic response. There were a lot of studies focusing on the evaluation of TIME, estimation of CD8 cells inside tumor to discriminate TIME is a common method (Sun et al. 2018). But biopsies are needed for the estimation of cells, so this approach is relatively complex, and the acquisition of biopsies could potentially lead to tumor metastasis. Compared with previous studies, we have employed CODEX technology that can simultaneously detect 17 immune-related biomarkers, which not only allows for a more comprehensive detection of immune microenvironment in tumors, but also avoids batch effects that may arise from multiple sections of immunohistochemistry. We also found that for patients with low RIS, they benefit more from anti-PD-1 immunotherapy. Therefore, for those patients, receiving anti-PD-1 immunotherapy after surgery might improve the prognosis.

Despite the above strengths, we also have some limitations in this study. First of all, due to limitations in technological, we did not have a large sample size in training cohort, further study with a larger sample size should be warranted in the future; Second, our study was retrospective study, a prospective study would be carried out to evaluate the potential value of RIS model for prognostic and drug effect prediction. Due to the inter-tumoral heterogeneity, TMA-based TIME evaluation cannot provide a global understanding of TIME status within the whole tumor nodule. Hence, multiple-spots biopsy might be used, and integrated analysis with radiomic image would be beneficial for establishing a more reliable and practical model. Non-viral HCC, especially  nonalcoholic steaohepatitis (NASH)-induced HCC is less responsive to immunotherapy (Pfister et al. 2021). In our study, the information of NASH-induced HCC patients has not been collected. In addition, HBV infection and liver cirrhosis are also associated with TIME in HCC (Lim et al. 2019; Yang et al. 2014; Zhang et al. 2023). We had not considered the potential effect of hepatitis B virus (HBV) infection and liver cirrhosis in our study. For primary HCC and advanced HCC patients, the TIME might be different, as well as different tumor size. In our future research, we will consider these factors and make up for the limitations of our study.

## Conclusion

In conclusion, we developed a noninvasive radiomic strategy nominated as RIS model which has been approved with higher accuracy to monitor TIME status, predict clinical outcome, and evaluate anti-PD-1 immunotherapy responsiveness for HCC patients. Further, we might investigate the radiomic signature for predicting and monitoring other types of immunotherapies.

### Supplementary Information

Below is the link to the electronic supplementary material.

Supplementary file1 (DOCX 1669 KB)

## Data Availability

The MRI images and clinical information from TCGA cohort can be accessed on the TCIA online database (https://www.cancerimagingarchive.net/, cohort TCGA LIHC) and GDC Portal (http://www.tcgaportal.org/, cohort TCGA LIHC). The CODEX and MRI images generated in this study are not publicly available due to patient privacy requirements but are available upon reasonable request from the corresponding authors. Other data generated in this study are available within the article and its supplementary data files.

## Abbreviations

AUC: Area under the curve
CD8: Cluster of differentiation 8
CI: Confidence interval
CODEX: Co-detection by indexing
CR: Complete response
CT: Computed tomography
CV: Coefficient of variation
EHBH: Eastern Hepatobiliary Surgery Hospital
FFPE: Formalin-fixed, paraffin embedded
HCC: Hepatocellular carcinoma
ICI: Immune checkpoint inhibitor
IS: Immunoscore
LASSO: Least absolute shrinkage and selection operator
MRI: Magnetic resonance imaging
NLR: Neutrophil-to-lymphocyte ratio
OS: Overall survival
PD-1: Programmed cell death 1
PD-L1: Programmed cell death ligand 1
PR: Partial response
PD: Progressed disease
RIS: Radiomic Immunoscore
RS: Radiomic Score
SD: Stable disease
TCGA: The Cancer Genome Atlas
TCIA: The Cancer Imaging Archive
TIME: Tumor immune microenvironment
TMA: Tissue microarray
