# Supplementary material for: A Noninvasive Approach to Evaluate Tumor Immune Microenvironment and Predict Outcomes in Hepatocellular Carcinoma
Source: Phenomics. 2023 Dec 8;3(6):549–64. doi: 10.1007/s43657-023-00136-8 (PMC10781918; doi:10.1007/s43657-023-00136-8)
Supplement: Supplementary file 1 — Supplementary file1 (DOCX 1669 KB) [file 43657_2023_136_MOESM1_ESM.docx]

**Supplementary Materials**

**Contents:**

- **Supplementary Methods**
- **Supplementary Figures**
- **Supplementary Tables**

**Supplementary Methods**

1. **Patients**

The overall study design is shown in Fig. S1. We retrospectively reviewed data from 274 patients with HCC in Eastern Hepatobiliary Surgery Hospital (EHBH), 25 patients from dataset of The Cancer Genome Atlas (TCGA) and 35 patients from Union Hospital Tongji Medical College Huazhong University of Science and Technology (Fig. S2). The patients in EHBH inclusion criteria were: histologically confirmed HCC; Enhanced MRI imaging performed <30 days before surgical resection; no preoperative chemotherapy; and complete information about clinicopathological characteristics and follow-up data available. The patients in TCGA cohort inclusion criteria were: histologically confirmed hepatocellular carcinoma (HCC); Enhanced magnetic resonance imaging (MRI) imaging performed before treatment; and complete information about clinicopathological characteristics and follow-up data available. The patients in immunotherapy cohort inclusion criteria were: histologically confirmed HCC; Enhanced MRI imaging performed before anti-PD-1 (anti-programmed cell death 1) immunotherapy; and complete information about clinicopathological characteristics and follow-up data available.

1. **CODEX FFPE tissue staining and fixation**

Formalin-fixed, paraffin embedded (FFPE) tissue blocks were retrieved from the tissue archive at the Institute of Pathology, Eastern Hepatobiliary Surgery Hospital, Shanghai, China. For the tissue microarray (TMA), tissues were selected from tumor region of FFPE tissue blocks. 1mm diameter cores was assembled. FFPE samples were cut into 3-um section.

The coverslip containing the tissue section was baked at 70℃ for 1 hour, deparaffinized in xylene, rehydrated in ethanol, then washed in ddH_2_O. After antigen retrieval, the coverslip was stained with antibody with 17-marker panel (see Supplementary Table 2) to a volume of 190ul and incubate for 3 hours at room temperature. Imaging of the (Co-detection by indexing) CODEX multicycle experiment was performed using an inverted fluorescence microscope (Keyence, Osaka, Japan; Model BZ-X710) equipped with a CFI plan Apo λ 20x/0.75 objective (Nikon, Tokyo, Japan), a microfluidics instrument (Akoya Bioscience, Menlo Park, CA, USA), and CODEX driver software (Akoya Bioscience, Menlo Park, CA, USA). The DAPI nuclear stain (Akoya Bioscience, #7000003) was acquired in the final cycle.

1. **Processing and Analysis of CODEX Data**

Raw image files were processed using CODEX Processer (Akoya Bioscience, Menlo Park, CA, USA). Seven-color overlay images with select markers were created in ImageJ. CODEX Multiplex Analysis Viewer (version 1.5.0.8) was used to analyze the protein expression inside each tumor.

1. **MR Acquisition and Image**

All magnetic resonance (MR) images were obtained from GE Optima MR360 1.5T equipped with an 8-channel abdominal coils. Patients were fasted for four hours before the scan. Gd-BOPTA (MultiHance, Bracco) with a total dose of 0.1mmoL/kg was injected into the median cubitus vein at a rate of 2.0 mL/s with a high-pressure syringe, followed by washing with 20mL of normal saline. Arterial, portal, and delayed scans were performed at 20-30 s, 50-60 s and 90-120 s after the injection of Gd- BOPTA, respectively. Fat-suppressed T2-weighted images, T1WI and FIESTA were collected. Detailed scanner and scan parameters can be found in Supplementary Table E1.

Feature extraction and image preprocessing were performed with the 3D Slicer software (version4.9.0; http://www.slicer.org). Images were resampled to a voxel size of 1 × 1 × 1 mm to standardize the voxel spacing; voxel intensity values were discretized by using a fixed bin width of 25 HU to reduce image noise and normalize intensities, allowing for a constant intensity resolution across all tumor images. We extracted 1223 radiomic features (14 shape features, 75 texture features, 18 first-order statistics, and 752 wavelet decompositions) from each three-dimensional segmentation, giving a total of 6115 features for every lesion.

A total of 6115 features were extracted from MRI images. We have tested different normalization method, including min-max normalization method and z-score normalization method. After testing, we chose min-max normalization method for its better performance in prediction the level of Immunoscore (IS).

1. **Construction of IS by using LASSO Cox Regression Model**

The least absolute shrinkage and selection operator method (LASSO) uses an L1 penalty to shrink some regression coefficients to exactly zero. We plotted the mean square error (MSE) versus log (λ), where λ is the tunning parameter for the LASSO logistic regression model. A value of λ= 0.052 with log (λ) = -2.957 was chosen by 10-fold cross-validation via the min criteria. Five features, CD68, HLA-DR, CD44, CD20, and CD31, with coefficients -9.022618, -1.661938, 8.106825, 2.089563, and 3.840371, respectively, were selected in the LASSO Cox regression model (Figure S3A). The status of TIME was evaluated by the following formula: Immunoscore = -9.023 * (CD68) – 1.662 ∗ (HLA-DR) + 8.107∗ (CD44) + 2.09 ∗ (CD20) + 3.84 ∗ (CD31). Patients were classified as high IS and low IS according to the optimum cutoff 0.672. We investigated the prognostic of the IS by using Kaplan-Meier survival analysis and log-rank test.

1. **Construction of Radiomic Immunoscore (RIS) using ridge regression model**

We used LASSO regression model with 10-fold cross validation. A value λ = 0.167 with log (λ) = -1.79 was chosen via the min criteria. The optimal tuning parameter resulted in five features shown in Supplementary Table 2. Then RIS was built by using a ridge regression model.

The “glmnet” package was used to perform the LASSO regression model and ridge regression model. The prediction ability of the model was assessed with the mean-squared error (MSE). The optimal cutoff value for RIS was determined by using Youden’s index in EHBH cohort 1, which maximized the sum of sensitivity and specificity. This cutoff was fixed and then applied to the validation cohorts.

1. **Construction of Radiomic Score (RS) using LASSO-logistic regression model**

RS was built using following formula: RS = 0.601 * (glrlm-ShortRunHighGrayLevelEmphasis) + 0.166 * (glrlm-GrayLevelNonUniformityNormalized) + 0.141 * (glcm-Imc1) - 0.474.

1. **Construction of clinical model using COX regression model**

The risk score of clinical model was built by 5 biomarkers: CEA (ug/L), AFP (ug/L), CA724 (U/ml), CA199 (U/ml) and CA125 (U/ml), using following formula: Risk Score = 0.167*(CEA) + 0.0003*(AFP) + 0.068*(CA724) + 0.02*(CA199) + 0.002*(CA125)

1. **Association with prognosis and anti-PD-1 immunotherapy**

We evaluated the RIS in three validation cohort: EHBH cohort 4, TCHA cohort and immunotherapy cohort. All MRI images were collected before treatment. In immunotherapy cohort, MRI images were collected before and after anti-PD-1 treatment.

1. **Integrated Nomogram**

An integrated nomogram was developed by using radiomics combined with clinical and pathologic features in EHBH cohort 1 and EHBH cohort 3. The integrated nomogram incorporated RIS, IS, gender, age, and the prognostic clinicopathologic risk factors (TNM stage, tumor number). To quantify the discrimination performance, Harrell’s concordance index (C-index) was measured. Calibration curves were generated to compare the predicted survival with the actual survival. “rms” packages were used to generate nomograms and calibration curves.

**Supplementary Figure Legends**

**Fig. S1 Workflow of overall study**

Using CODEX data from EHBH cohort 1, we constructed Immunoscore (IS), IS was found to be associated with overall survival, this association was further validated in an independent validation cohort (EHBH cohort 2). Additionally, a radiomic model (RIS model) was developed based on MRI images to predict IS, and the RIS was validated in two independent cohorts for its ability to predict prognosis. Furthermore, the RIS was found to be associated with immunotherapy response in the Immunotherapy cohort.

**Fig. S2 Flow chart of patient inclusion**

487 patients were recruited in our study, according to the enrollment criteria, a total of 301 HCC patients were included in our study. Among them, 241 patients were from Eastern Hepatobiliary Surgery Hospital, 25 patients were from TCGA database, 35 patients treated with anti-PD-1 immunotherapy were from Union Hospital Tongji Medical College Huazhong University of Science and Technology.

**Fig. S3 Protein expression in patients of the high IS and low IS group**

The expression of CD20, CD31, CD44, CD163 and PD-1 was significantly higher in the high IS group than in low IS group.

**Fig. S4 The association of IS with TNM stage and metastasis**

**a** IS of patients in stage III and stage IV was significantly higher than patients in stage I and stage II. **b** In patients with metastasis, the IS were significantly higher than non-metastasis patients.

**Fig. S5 Model selection and construction of RIS**

**a** Tuning parameter (λ) selection in the LASSO method used via 10-fold cross-validation. **b** LASSO coefficient profiles of the 751 texture features. **c** Performance of different models in the testing cohort, including the logistic regression model, SVM, ridge regression model, random forest, XGBoost and linear regression model, area under the curve (AUC) was used to evaluate the performance of model in the testing cohort (EHBH cohort 3).

**Fig. S6 Nomogram based on RS and clinicopathological factors and their calibration curve**

Nomogram predicting survival of HCC patients were established based on RS and clinicopathological factors.

**Fig. S7 The association between TNM stages and responses to anti-PD-1 immunotherapy in Immunotherapy Cohort.**

Number of patients in different stages and their responses to anti-PD-1 immunotherapy cohort.

**Supplementary Figures**


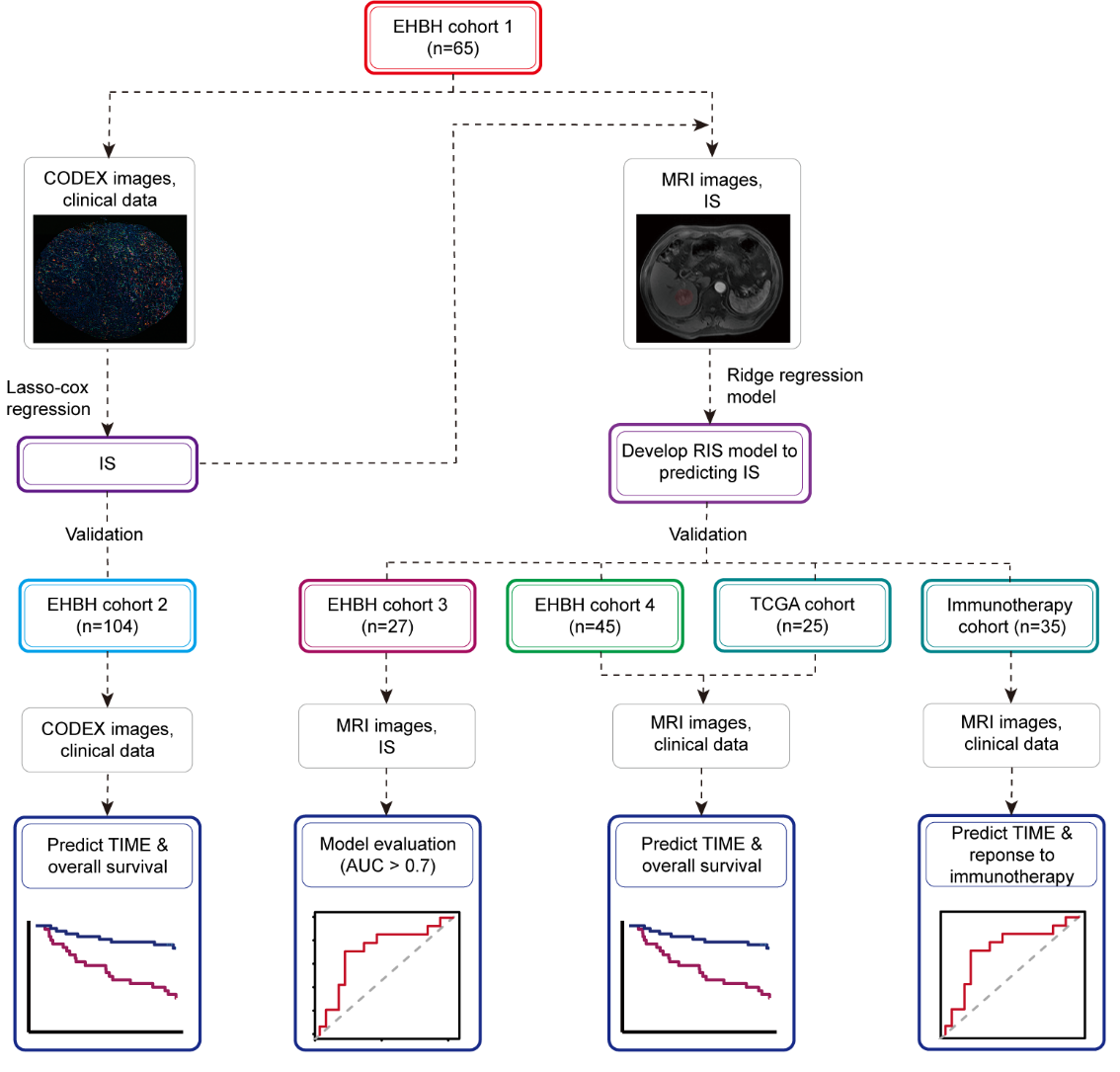


**Fig. S1 Workflow of overall study**


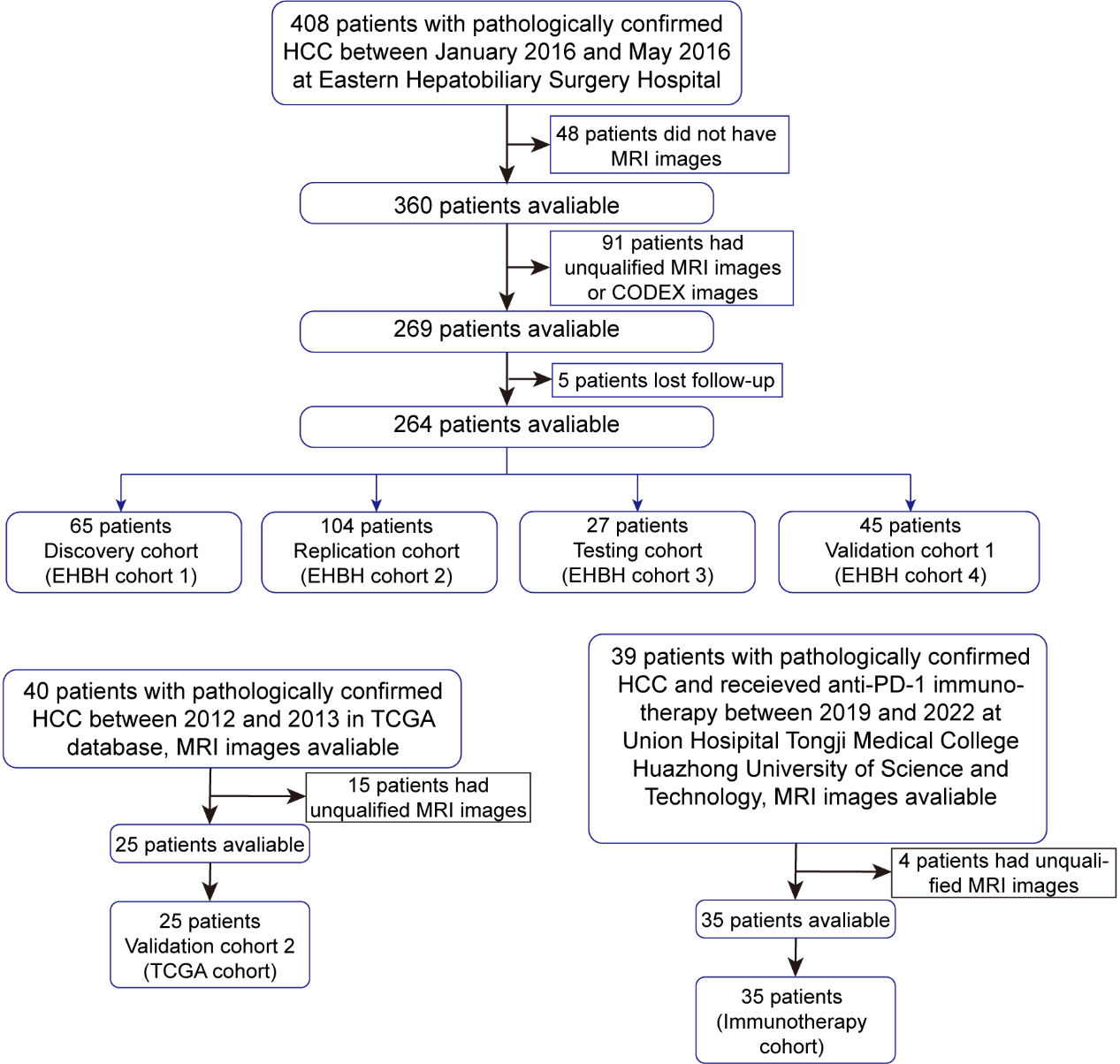


**Fig. S2 Flow chart of patient inclusion**


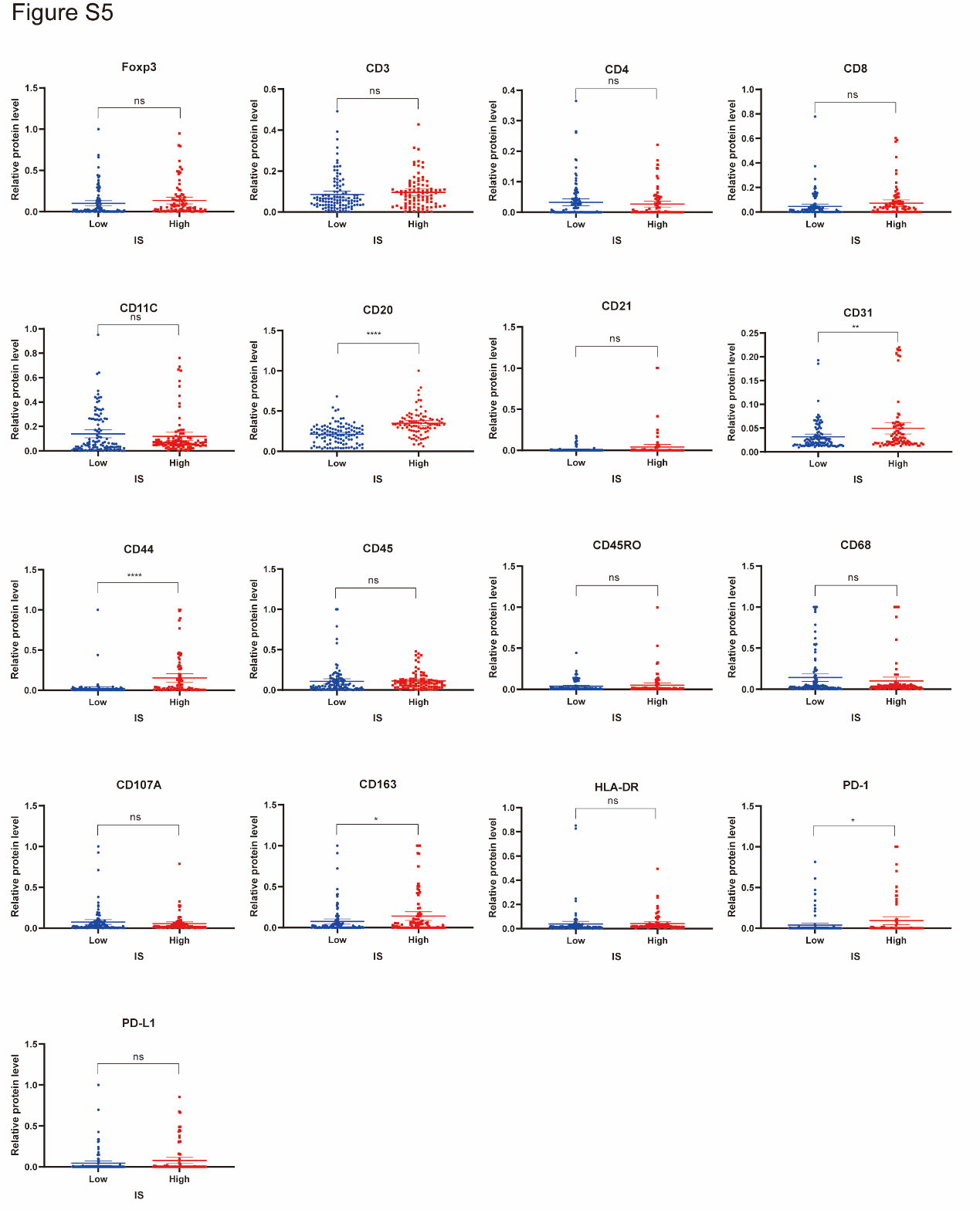


**Fig. S3 Protein expression in high IS and low IS group**


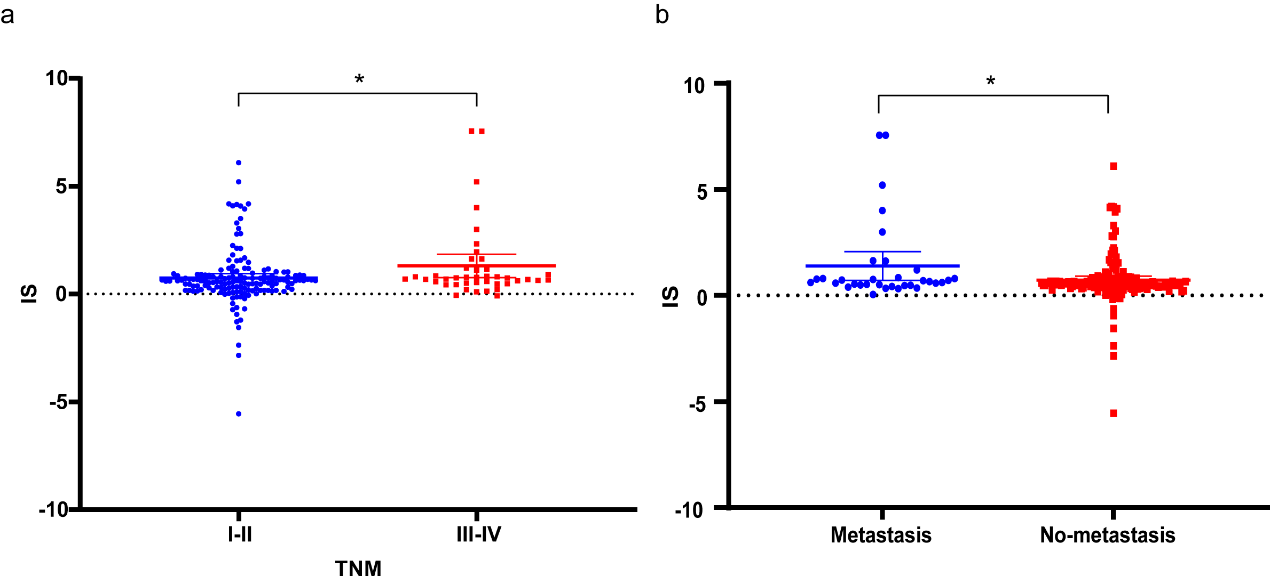


**Fig. S4 The association of IS with TNM stage and metastasis**


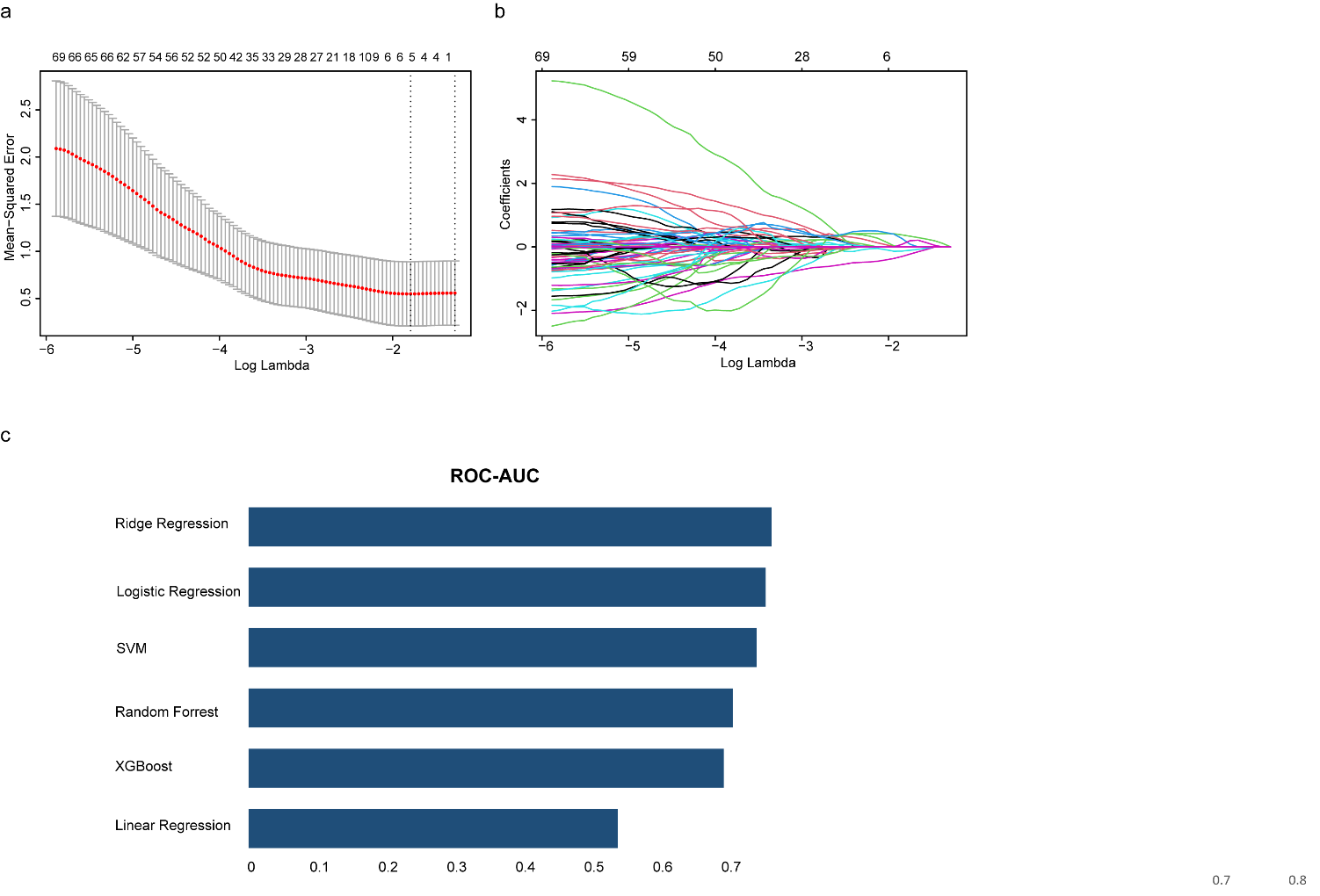


**Fig. S5 Model selection and construction of RIS**


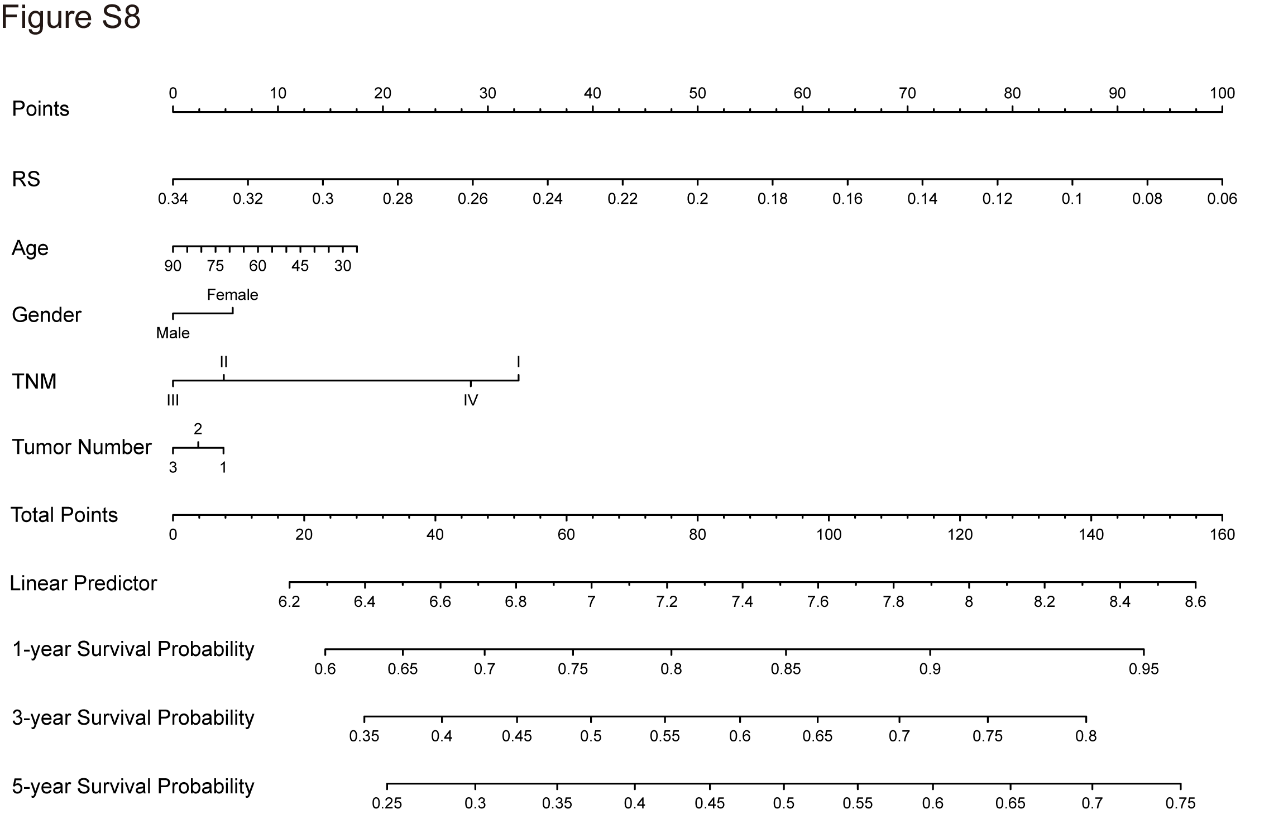


**Fig. S6 Nomogram based on RS and clinicopathological factors and their calibration curve**


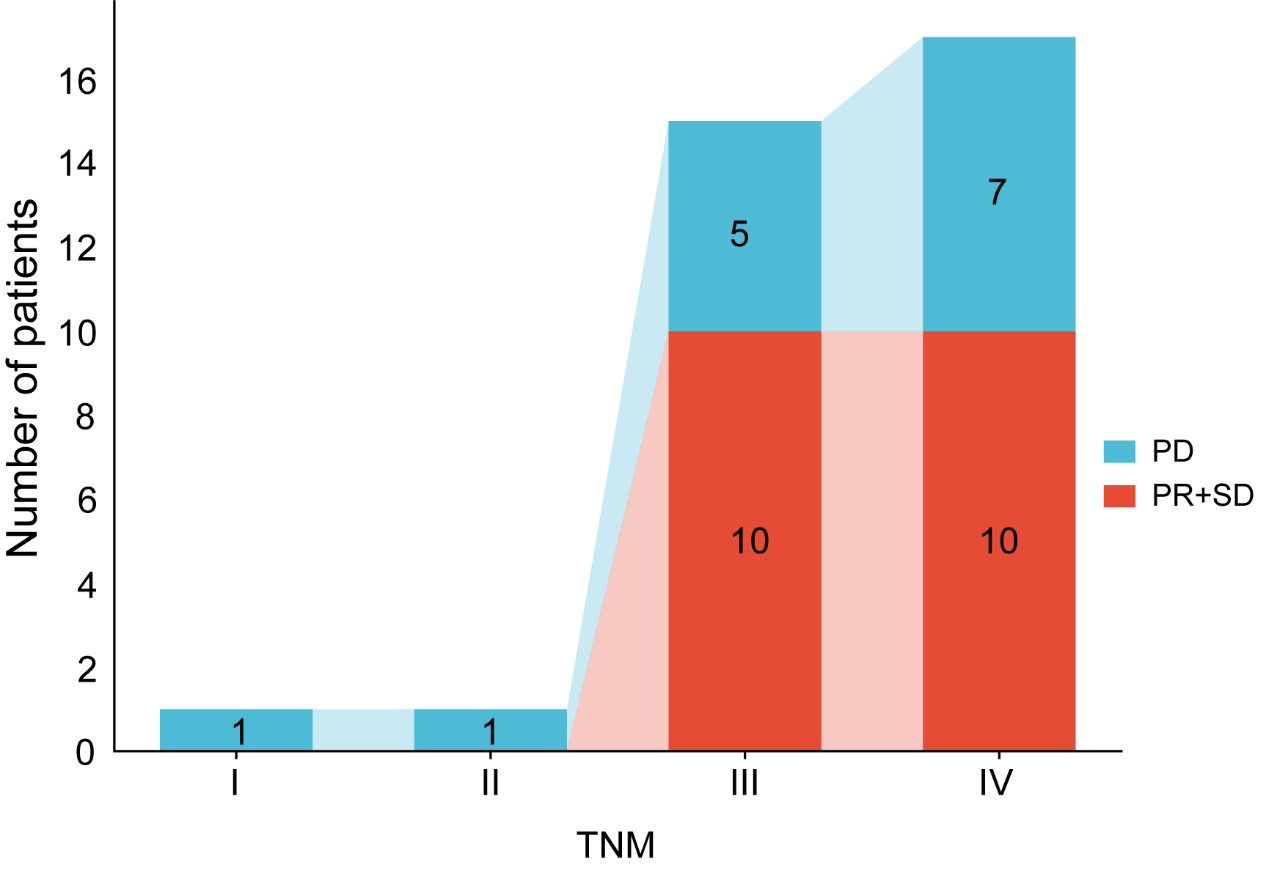


**Fig. S7 The association between TNM stages and responses to anti-PD-1 immunotherapy in Immunotherapy Cohort.**

**Supplementary Tables**

**Table S1** Treatment information of patients in Immunotherapy cohort

| Patient No | Treatment | HBV infection | Liver cirrhosis | TNM stage | Tumor size (cm) | Response |
| --- | --- | --- | --- | --- | --- | --- |
| 1 | Anti-PD-1+radiotherapy | Yes | Yes | Ⅲ | 8.6 | PR |
| 2 | Anti-PD-1+radiotherapy | No | No | Ⅳ | 5.6 | PR |
| 3 | Anti-PD-1+Sorafenib | Yes | No | Ⅲ | 10.4 | PD |
| 4 | Anti-PD-1+TKI | Yes | Yes | Ⅳ | 7.1 | PR |
| 5 | Anti-PD-1+radiotherapy | Yes | Yes | Ⅲ | 12.0 | SD |
| 6 | Anti-PD-1+radiotherapy | Yes | Yes | Ⅳ | 6.73 | PD |
| 7 | Anti-PD-1 | Yes | Yes | Ⅳ | 8.0 | PR |
| 8 | Anti-PD-1 | No | Yes | Ⅳ | 12.0 | PR |
| 9 | Anti-PD-1+TKI | No | No | Ⅲ | 4.0 | PD |
| 10 | Anti-PD-1+TKI | No | No | Ⅲ | 6.8 | SD |
| 11 | Anti-PD-1+TKI | No | No | Ⅳ | 4.0 | PR |
| 12 | Anti-PD-1+Stivarga | Yes | No | Ⅲ | 1.9 | PD |
| 13 | Anti-PD-1+Apatinib | Yes | Yes | Ⅳ | 2.8 | PD |
| 14 | Anti-PD-1 | Yes | No | Ⅳ | 5.1 | PD |
| 15 | Anti-PD-1+TKI | Yes | No | Ⅳ | 15.8 | PD |
| 16 | Anti-PD-1 | No | Yes | Ⅳ | 14.0 | PD |
| 17 | Anti-PD-1 | Yes | Yes | Ⅲ | 6.0 | PD |
| 18 | Anti-PD-1 | No | No | Ⅳ | 13.0 | PD |
| 19 | Anti-PD-1 | No | No | Ⅰ | 1.7 | PD |
| 20 | Anti-PD-1+Lenvatib | Yes | Yes | Ⅳ | 9.0 | PD |
| 21 | Anti-PD-1 | Yes | Yes | Ⅳ | 9.0 | PR |
| 22 | Anti-PD-1 | Yes | Yes | Ⅲ | 20.0 | SD |
| 23 | Anti-PD-1+Apatinib | Yes | Yes | Ⅲ | 2.7 | SD |
| 24 | Anti-PD-1+Apatinib | Yes | Yes | Ⅱ | 2.3 | PD |
| 25 | Anti-PD-1+Fruquintinib | No | Yes | Ⅲ | 6.0 | SD |
| 26 | Anti-PD-1+Lenvatinib | No | No | NA | NA | SD |
| 27 | Anti-PD-1 | No | Yes | Ⅲ | 17.0 | PD |
| 28 | Anti-PD-1+Apatinib | Yes | No | Ⅲ | 14.0 | PR |
| 29 | Anti-PD-1 | No | Yes | Ⅳ | 4.6 | PR |
| 30 | Anti-PD-1+CapeOX+radiotherapy | Yes | Yes | Ⅲ | 8.0 | PR |
| 31 | Anti-PD-1+ Apatinib | Yes | Yes | Ⅲ | 5.9 | PR |
| 32 | Anti-PD-1+Lenvatinib | Yes | Yes | Ⅳ | 8.0 | SD |
| 33 | Anti-PD-1+Bevacizumab | Yes | No | Ⅳ | 6.8 | PR |
| 34 | Anti-PD-1+Lenvatinib | No | No | Ⅳ | 5.8 | SD |
| 35 | Anti-PD-1 | Yes | Yes | III | 10.7 | SD |

PR: Partial Response, PD: Progressive Disease, SD: Stable Disease

**Table S2** Antibody panel of CODEX

| Antibody | Antibody-Barcode | Fluo | Company | Identifier |  |
| --- | --- | --- | --- | --- | --- |
| Foxp3 | Foxp3-BX020 | ATTO-550 | Biolegend | BX020-ATTO550 |  |
| PD-L1 | PD-L1-BX090 | ATTO-550 | Abcam | BX029-ATTO550 | |
| PD-1 | PD-1-BX035 | ATTO-550 | Abcam | BX035-ATTO550 | |
| CD163 | CD163-BX041 | ATTO-550 | Abcam | BX041-ATTO550 | |
| CD45 | CD45-BX021 | CY5 | Abcam | BX049-AF750 | |
| CD45RO | CD45RO-BX017 | ATTO-550 | Akoya | 425003 | |
| CD107A | CD107A-BX006 | CY5 | Akoya | 4350001 | |
| CD21 | CD21-BX032 | ATTO-550 | Akoya | 4450027 | |
| CD68 | CD68-BX015 | CY5 | Akoya | 4350019 | |
| CD8 | CD8-BX026 | ATTO-550 | Akoya | 4250012 | |
| CD3 | CD3-BX045 | CY5 | Akoya | 4450030 | |
| CD4 | CD4-BX003 | CY5 | Akoya | 4350018 | |
| CD11C | CD11C-BX024 | CY5 | Akoya | 4350020 | |
| HLA-DR | HLA-DR-BX003 | CY5 | Akoya | 4450029 | |
| CD44 | CD44-BX005 | ATTO-550 | Akoya | 4250002 | |
| CD20 | CD20-BX007 | ATTO-750 | Akoya | 4450018 | |
| CD31 | CD31-BX001 | ATTO-750 | Akoya | 4450017 | |

**Table S3** Predictive radiomic features

| **Imaging sequence** | **Feature name** | **Future type** |
| --- | --- | --- |
| T1 | MCC | (Wavelet-LHH)-glcm |
| T1 | Contrast | (Wavelet-HLH)-glcm |
| T1 | GrayLevelVariance | (Wavelet-HHL)-glrlm |
| D3 | RunVariance | (Wavelet-LHH)-glrlm |
| D3 | RunVariance | (Wavelet-HLH)-glrlm |

**Table S4** Multivariate Cox regression analyses for overall survival in EHBH cohort 1

| Variables | Overall survival | |
| --- | --- | --- |
|  | HR (95% CI) | p |
| IS (low versus high) | 0.34 (0.17, 0.7) | 0.0033 |
| Age (<=60 versus >60) | 0.81 (0.4, 1.6) | 0.55 |
| Gender (Male versus Female) | 0.98 (0.34, 2.8) | 0.97 |
| Stage (I + II versus III + IV) | 0.7 (0.34, 1.5) | 0.35 |

**Table S5** Multivariate Cox regression analyses for overall survival in EHBH cohort 1

| Variables | Overall survival | |
| --- | --- | --- |
|  | HR (95% CI) | p |
| RIS (low versus high) | 0.42 (0.18, 0.99) | 0.048 |
| Age (<=60 versus >60) | 0.99 (0.48, 2.1) | 0.99 |
| Gender (Male versus Female) | 0.88 (0.3, 2.5) | 0.81 |
| Stage (I + II versus III + IV) | 0.24 (0.076, 0.73) | 0.013 |

**Table S6** Univariate association of IS, RIS, clinicopathological characteristics with overall survival in EHBH cohort 1

| Variables | Overall survival | |
| --- | --- | --- |
|  | HR (95%CI) | p |
| IS (low versus high) | 0.329 (0.164, 0.659) | 0.0017 |
| RIS (low versus high) | 0.373 (0.162, 0.858) | 0.02 |
| Age (years) (≤60 versus >60) | 0.8 (0.408, 1.572) | 0.518 |
| Gender (male versus female) | 0.905 (0.319, 2.571) | 0.852 |
| Distant metastasis (metastasis versus non-metastasis) | 0.903 (0.408, 1.997) | 0.801 |
| Stage (I,II versus III,IV) | 0.547 (0.269, 1.111) | 0.095 |

**Table S7** Univariate association of IS, clinicopathological characteristics with overall survival in EHBH cohort 2

| Variables | Overall survival | |
| --- | --- | --- |
|  | HR (95%CI) | p |
| IS (low versus high) | 0.548 (0.306, 0.982) | 0.043 |
| Age (years) (≤60 versus >60) | 0.809 (0.449, 1.456) | 0.479 |
| Gender (male versus female) | 1.223 (0.519, 2.881) | 0.645 |
| Distant metastasis (metastasis versus non-metastasis) | 2.037 (1.054, 3.939) | 0.034 |
| Stage (I,II versus III,IV) | 0.527 (0.278, 1.0) | 0.05 |

**Table S8** Univariate association of IS, RIS, clinicopathological characteristics with overall survival in EHBH cohort 3

| Variables | Overall survival | |
| --- | --- | --- |
|  | HR (95%CI) | p |
| IS (low versus high) | 0.366 (0.131, 1.022) | 0.055 |
| RIS (low versus high) | 0.587 (0.203, 1.698) | 0.326 |
| Age (years) (≤60 versus >60) | 1.668 (0.605，4.60) | 0.323 |
| Gender (male versus female) | 1.262 (0.285, 5.580) | 0.759 |
| Distant metastasis (metastasis versus non-metastasis) | 0.483 (0.165, 1.410) | 0.183 |
| Stage (I,II versus III,IV) | 0.632 (0.202, 1.980) | 0.431 |
| Tumor number (≥2 versus <2) | 2.642 (0.583, 11.983) | 0.208 |

**Table S9** Univariate association of RIS, clinicopathological characteristics with overall survival in EHBH cohort 4

| Variables | Overall survival | |
| --- | --- | --- |
|  | HR (95%CI) | p |
| RIS (low versus high) | 0.422 (0.157, 1.134) | 0.087 |
| Age (years) (≤60 versus >60) | 1.407 (0.614, 3.223) | 0.419 |
| Gender (male versus female) | 2.563 (0.346, 19.004) | 0.357 |
| Stage (I, II versus III, IV) | 1.228 (0.289, 5.226) | 0.781 |

**Table S10** Univariate association of RIS, clinicopathological characteristics with overall survival in Combined cohort (TCGA cohort plus EHBH cohort 4)

| Variables | Overall survival | |
| --- | --- | --- |
|  | HR (95%CI) | p |
| RIS (low versus high) | 0.516 (0.233, 1.143) | 0.103 |
| Age (years) (≤60 versus >60) | 1.244 (0.622, 2.491) | 0.537 |
| Gender (male versus female) | 1.619 (0.626, 4.187) | 0.320 |
| Distant metastasis (metastasis versus non-metastasis) | 1.082 (0.148, 7.920) | 0.938 |
| Stage (I,II versus III,IV) | 0.734 (0.318, 1.697) | 0.470 |
